# Clinical, molecular and drug sensitivity pattern of mycobacterial isolates from extra-pulmonary tuberculosis cases in Addis Ababa, Ethiopia

**DOI:** 10.1186/s12879-015-1177-4

**Published:** 2015-10-26

**Authors:** Workneh Korma, Adane Mihret, Jemal Hussien, Richard Anthony, Mekuria Lakew, Abraham Aseffa

**Affiliations:** Institute of Biotechnology, Addis Ababa University, P.O. Box 62720, Addis Ababa, Ethiopia; Armauer Hansen Research Institute, P.O.Box 1105 Addis Ababa, Ethiopia; Royal Tropical Institute, Meibergdreef 39, 1105 AZ Amsterdam, The Netherlands; Department of Microbial, Cellular and Molecular, Addis Ababa University, P.O. Box 1176 Addis Ababa, Ethiopia

**Keywords:** Extra pulmonary tuberculosis, Strain variation, Addis Ababa, Drug resistance

## Abstract

**Background:**

In conjunction with the spread of HIV infection, tuberculosis (TB) remains a major cause of illness and death worldwide. The Ethiopian national report reveals that extra pulmonary tuberculosis is on the rise and that case detection rate is exceeding that of smear positive or negative cases in many parts of the country. Different studies indicated that host and/or pathogen related factors are associated with the rise of extra pulmonary cases. However, the reason for this is not clearly known in our setting.

**Methods:**

Specimens were taken from clinically suspected extra pulmonary patients and confirmed by cytology, histopathology and culture. Deletion typing and Spoligotyping was utilized to identify the strains. The isolates were then assigned to lineage using conformal Bayesian network (rules model) algorithm and dendrograms were drawn using UPGMA methods. In addition, drug sensitivity test was done using the indirect proportion and 24 well plate methods.

**Results:**

Out of the 200 clinically suspected extra pulmonary tuberculosis patients, 106 (53 %) were between 15 and 35 years of age and 167 (83.5 %) were new while 33 (16.5 %) were retreatment cases. The culture yield was 29.5 % (59). Of these only one was *M. bovis* and 58 were *M. tuberculosis* strains with 31 different spoligotype patterns grouped into seven clusters. The largest cluster (ST53) comprised 12 (20.3 %) isolates. There was higher clustering of CAS isolates in TBLN than in any other form of extra pulmonary tuberculosis cases. Resistance to rifampicin was higher (22 %) than that for INH, STM and EMB (8.1 %, 5 % and 3 % respectively). Out of the 37 isolates tested for resistance, only 2 isolates were resistant for both STM and INH and no MDR strain was found.

**Conclusions:**

There is an ongoing active recent transmission among extra pulmonary tuberculosis in the study areas as shown by the presence of clusters. Although no MDR case was observed, there is a risk of emergence of MDR as noted from the high proportion of resistance to rifampicin. Detailed study at population level is recommended to monitor its trend.

## Background

In conjunction to HIV, tuberculosis remains a major global health problem. It causes ill-health among millions of people each year and ranks as the second leading cause of death from an infectious disease worldwide, after the human immunodeficiency virus (HIV). According to the latest estimates, globally 8.6 million people develop TB and 1.3 million died from the disease in 2012. The South-East Asia and Western Pacific Regions collectively accounted for 58 % of the world’s TB cases in 2012 and the African region had approximately one quarter of the world’s cases [1].

In Ethiopia, the number of caseloads reported in all forms of TB in 2012 was 143 503 which makes it one of the largest reported cases in the world [1]. There are 22 high burden countries (HBCs), which accounts 81 % of the estimated number of new TB cases (all forms) arising worldwide each year. Of these Ethiopia ranks in the 7^th^ position after India, China, Indonesia, Pakistan and South Africa. According to WHO (2013) estimate; the prevalence, incidence and mortality in all forms of TB were 224, 247 and 18 per 100,000 respectively [1] Based on the national surveillance data in the year 2010/11 a total of 159,017 TB cases were notified and the proportion of new smear-positive, smear negative and Extra Pulmonary Tuberculosis (EPTB) among all new cases is 32.7 %, 34.8 %, and 32.5 % respectively.

With the advent of HIV/AIDS the incident rate of EPTB is also on the rise and becomes worldwide problem [2–5]. The disease is highly pronounced in countries where HIV infection and incident TB rate is higher. Accordingly, EPTB accounts for 15 to 20 % of all forms in people who do not have HIV but 53 to 62 % in HIV co-infected individuals [6,7]. The case notification of extra pulmonary tuberculosis is in Ethiopia is also higher and almost equal to that of smear positive and negative pulmonary tuberculosis cases with a percentage of 33 each [8].

The development of DNA finger printing (genotyping) of *Mycobacterium tuberculosis* isolates has provides a better insight in understanding of the transmission of tuberculosis [9]. Genotyping outcome of the isolates together with epidemiological data assists researchers to identify recent transmission and trace outbreaks. The techniques can be utilized as a tool for epidemiological studies to determine the overall diversity of *Mycobacterium tuberculosis* strain that can help to address important epidemiological questions such as the origin of an infection in patient’s household or community, and the spread. It also assists in an early detection of organisms with acquired drug resistance [9, 10]. Although a number of molecular techniques are available, spoligotyping is selected for a number of reasons. It is relatively simple, rapid and good for strains with low copy numbers of IS*6110*. Moreover it helps to identify and type strains simultaneous [11].

It has been indicated that strain variation associated with clinical manifestation of tuberculosis [12, 13] and explained that some strain possess superior ability than others and becomes highly virulent to causes extra thoracic tuberculosis and disseminated out of the lung [12, 14–16]. Moreover, recent studies reported the variation in the genetic diversity of tuberculosis causing pulmonary and extra pulmonary tuberculosis in different cities of Ethiopia [17–22]. However, data on extra pulmonary tuberculosis in Addis Ababa was not reported. Therefore, in this study we tried to evaluate the genetic diversity, clinical manifestation and drug sensitivity of mycobacterial isolates causing extra pulmonary tuberculosis is Addis Ababa city.

## Materials and methods

### Study population

A total of 200 clinical suspects of extra pulmonary TB patients that are referred and/or admitted in four referral hospitals found in Addis Ababa, Ethiopia (Black lion, St. Peter, St. Paul, and ALERT hospitals) from April 2012 to July 2013 and gave their written consent were considered. Using Pre structured questionnaires demographic, epidemiological and clinical data were collected. Patients were informed about the objectives and benefits of the study and who written consents were considered for the study. Children under 18 years of age were required to have guardian’s or parent’s informed consent and children between 12–18 years were requested for assent. The ethical approval was obtained both from the national and AHRI/ALERT Ethics Review committee.

### Specimen collection and processing

Specimens from extra pulmonary sites were collected by experienced health professionals (Medical doctors, Health Officers, Nurses) following standard procedure. Various fluids such as pleural, peritoneal and synovial fluids will be collected in sterile containers with anticoagulant. Tissue specimens were collected without fixatives. Where urogenital tuberculosis is suspected, three consecutive early morning urine samples without preservatives were taken. All clinical specimens were immediately transported to AHRI laboratory in an icebox.

Specimens from non-sterile sites were decontaminated by N-acetyl-L-Cycteine sodium hydroxide techniques and the sterile ones such as cerebrospinal, pleural, ascetic and Synovial fluids, were directly centrifuged at 3000 rpm for 15 min without decontamination [23]. The specimens was then be examined for AFB and inoculated on to Lowenstein Jensen medium (LJ medium) and then incubated at 37 °C, 7%CO_2_ for 6–8 weeks in slanted position as described on modified petroff’s method [24].

### Drug susceptibility test

The culture positive isolates were tested for drug sensitivity against first and second line anti-tubercular drugs following 24-well plate methods as described [25].

Stock solution for the first line drugs: INH, RIF, ETH and STR, (Sigma, St. Louis, USA) were prepared at 1 mg/ml concentration using distilled water for all but DMSO for rifampicine. It was then filter sterilized through 0.2 μm size filter and stored at -20C^o^ until used.

Nineteen gram of 7H10 Middle brook agars was dissolved (Becton Dickinson and Company) in 900 ml of distilled water at 50–60 °C temperature and 5 ml glycerol (BDH Laboratory Supplies, England) was added and autoclaved at 120 °C for 15 min. one hundred milliliter (10 %) Oleic acid-dextrose-catalase (OADC, Becton Dickinson and Company) was mixed after cooling it to 50 to 60 °C.

Working drug concentrations of the drugs including the critical concentration (Table 1) were mixed with sterile 7H10 media and dispensed in 3 ml. volume in 24 well plates. Two wells with drug free media were used as negative controls.

**Table 1:** Test concentrations of Anti-Tb drugs

Drug types	Test concentrations				Solvents
INH	0.064	0.125	0.2	1.0	dH_2_O
RIF	0.25	1.0			DMSO
EMB	1.0	2.0	5.0	8.0	dH_2_O
STM		2.0			dH_2_O

The red ones indicated critical concentrations

Drug sensitivity test was done based on modified proportion method in 24-well DST plate [25]. Determination of the susceptibility of isolates to anti‐mycobacterial drugs was done by standard indirect drug susceptibility test. All the isolates were tested for INH, RIF, EMB, STR, PAS, KAM and OFL resistance following the standard protocol [25].

A loop from culture was suspended in (ependorff) distilled water and two concentrations: McFarland turbidity 1 and 1:100 dilution of it were prepared. Ten μl from McFarland turbidity 1 suspension was inoculated to 22 wells with drug incorporated media and two drug free control media were inoculated with the two dilutions: McFarland turbidity 1 and 1:100 dilution. The plates were then incubated at 5 % CO2 concentration and 35oC temperature for 3–4 weeks and checked for growth at week intervals. Growth at concentrations ≥ critical was considered as resistant.

### HIV testing

Screening for HIV was done for only patients with unknown HIV status according to the Ethiopian National Guide lines. If the patient is found be positive, he/she was referred to the appropriate heath facilities rendering HIV care and follow up according to national guide lines [26].

### Species and strain identification

Species identification was done by using molecular methods. Molecular identification of the isolates was done based on the absence or presence of mycobacterium tuberculosis complex chromosomal region of difference (RD) deletion loci. The genome of the isolates was analyzed by multiple PCR assay for absence or presence of RD by using RD9 as previously described a [27].

Spoligotyping was employed for strain identification using commercially available kits using a commercially available kit (Isogen Bioscience BV, Maarssen, the Netherlands). The spacer sequences are interspersed between repetitive sequences and are variably present or absent in a given MTBC isolate. Examination of 43 of these unique spacer sequences results in a strain-specific fingerprint. The method is based on CRISPR (Clustered Regularly Interspaced Short Palindromic Repeats) region also called (direst repeat) DR region of Mycobacterium tuberculosis using the primers DRa (5’GGTTTTGGGTTTGAACGAC3’) and DRb (5’CCGAGAGGGGACGGAAAC3’) [28]. This direct region was amplified by using the DRa and DRb primers. The amplified DNA was hybridized to inter DR spacer oligonucleotides which are covalently bound to the membrane. Subsequently the amplified DNA was then hybridized to a set of 43 oligonucleotide probes by reverse blotting. The membrane then incubated with streptavidin peroxidase conjugate. Finally enhanced chemo luminescence detection system (Amersham, Little Chalfont, United Kingdom) was used and absence or presence of spacers was detected by visualizing the black spots developed on the membrane film.

### Database comparison

The spoligotyping patterns obtained were entered to the international spoligotyping database, SpolDB4.0 (http://www.pasteur-guadeloupe.fr:8081/SITVIT_ONLINE/) and assign in to the existing SIT (Spoligotype International Type) number. The spoligotypes not earlier described (orphans), not found in SpolDB4 data base, were also assigned to families and sub lineage using SPOTCLUST ID web based algorithm model SpolDB3 tanking the highest probability percentage (99 %) (http://tbinsight.cs.rpi.edu/run_spotclust.html).

Phylo-genetic lineages of Mycobacterium tuberculosis complex were done using lineages: four http://tbinsight.cs.rpi.edu/run_tb_lineage.html insight/tblineage.html web. Finally relationships among strains were also analyzed by drawing dendrgram using Unweighted Pair Group Method with Arithmetic mean (UPGMA) (http://genomes.urv.cat/UPGMA/).

## Result

### Demographic characteristics

Out of the 200 EPTB suspects who came to the four referral hospitals from different parts of the country within the study period, 94 (47 %) were males and 106 (53 %) were females. Their age ranged from 1 to 79 years with mean age of 25.91 (±15.67) years. However, the majority of them, 106 (53 %), were between 15 and 35 years. Most, 167 (83.5 %), were new and 33 (16.5 %) retreatment groups. Among the study participants, 55.1 % were married, 28.1 % single, 7.5 % divorced and 32 % underage (Table 1).

### Clinical presentation of patients

**Fig. 1:**
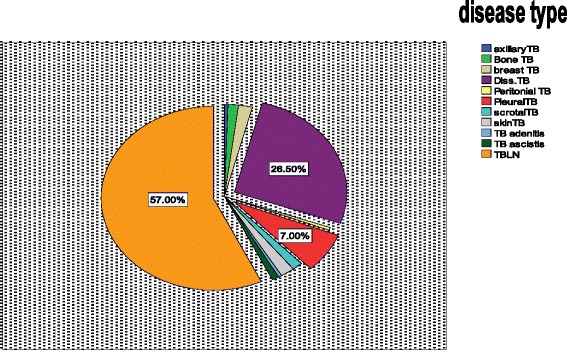
Pie chart of EPTB suspected cases

Based on the clinical presentation, 91.5 % of the enrolled patients fall into three major disease categories: 116 (58 %) tuberculous lymphadenitis or lymph node tuberculosis (TBLN), 53 (26.5 %) disseminated tuberculosis (DTB), 14 (7 %) pleural tuberculosis (PLTB) and the rest 9.5 % were mixes of scrotal, skin, peritoneal, leg, breast and peritoneal TB (Fig 1). Of the total sample population, 62 (31 %) were HIV positive, 123 (61.5 %) negative and 15 (7.5 %) of an unknown status. The 62 HIV positive patients were also categorized as TBLN [29], DTB [25], and, PLTB [3], and axial, bone and peritoneal TB (1 each) (Fig. 1). The highest HIV positive prevalence was in the age group 25–35 and among married subjects. In addition, of the total seventy five (37.5 %) were alcohol consumers and 15 (7.5 %) diabetic patients (Table 2).

**Table 2:** Demographic characteristics of EPTB patients

Variables		HIV result			P	*Χ* ^2^	% of HIV + ves
		+Ve	-Ve	unknown			
Age category	<5	3	17	1			4.84
	5-15	5	22	1			8.06
	15-25	8	38	6			12.90
	25-35	25	25	4	0.001	30.31	40.32
	35-45	14	12	1			22.58
	>45	7	8	3			11.29
Sex	Male	29	57	6			46.77
	Female	33	66	9	0.88	0.238	53.22
	Married	34	43	6			54.83
Marital Status	Unmarried	13	31	6			20.97
	Divorced/Widow/er	6	3	1	0.004	18.91	9.68
	Under age	9	46	2			14.52
Alcohol consumption	Yes	31	40	4			50
	No	31	83	11	0.045	6.186	50
Case type	New	40	112	15			64.52
	Retreatment	21	11	0		0	33.87
Diabetes Mellitus	Yes	5	8	2			8.07
	No	57	115	13			91.94

**Fig. 2:**
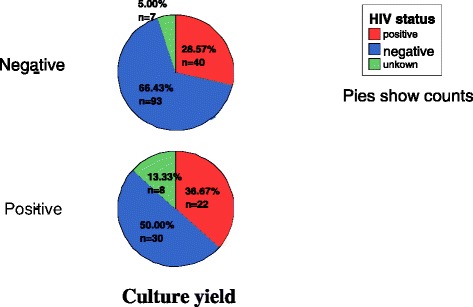
culture result Vs HIV positivity

The data also showed that from those confirmed by culture as EPTB cases, 36.7 % were HIV co-infected (Fig. 2)

.

### HIV and EPTB

Considering samples resulting culture positive as confirmed extra-pulmonary TB, we tried to assess it’s the relationship with HIV. As a result, the chi square test result shows that there is a significant relationship between HIV and extra-pulmonary tuberculosis with a p value =0.037 and *χ*2 = 6.620 (Table 3).

**Table 3:** Association between HIV and EPTB

	Value *χ*2	Asymp. Sig.(2-sided)
Pearson Chi-Square	6.620	.037
Linear-by-Linear Association	.001	.979
N of Valid Cases	200

**Table 4:** Spoligo patterns and family assignment of Extra-pulmonary tuberculosis isolates

No.	Spoligotype octal/binary format	Shared type number	Lineage using conformal Bayesian network (rules model)	sub lineage on SpolDB4	Spotclust ID based on SpolDB3 model	Spotclust probability	No.of isolates	%
1	111111111113771	1	East Asian (Beijing)	Beijing/W lineage	Beijing family		1	1.69
2	111111117761771	4	East-Asian	LAM3			1	1.69
3	700000007177771	910	Indo-Oceanic	U	Family 36		3	5.08
4	777000377760771	149	Euro-American	T3			6	10.1
5	777777777760771	53	Euro-American	T1			12	20.3
6	777777777720771	50	Euro-American	H3			1	1.69
7	777777777760751	612	Euro-American	T1			1	1.69
8	777775777760731	584	Euro-American	T1			1	1.69
9	777737777760771	37	Euro-American	T1			4	6.77
10	577777607760771	866	Euro-American	LAM9			1	1.69
11	777777777420771	777	Euro-American	H3			1	1.69
12	777777754020771	883	Euro-American	H1			1	1.69
13	777777774020771	47	Euro-American	H1			1	1.69
14	777737770000000	56	Euro-American	U	Family34/ EAI1/	0.99	1	1.69
15	703777740003171	25	East-African Indian (CAS)	CAS			5	8.47
16	703377400001771	21	East-African Indian (CAS)	CAS			3	5.08
17	777777377760771	40	Euro-American	T4			1	1.69
18	777737777420771	35	Euro-American	H4			1	1.69
19	603777740003771	952	East-African Indian (CAS)	CAS			1	1.69
20	577777002060771	788	Euro-American	T1			1	1.69
21	007777707760771	1889	Euro-American	U	LAM9	0.688	1	1.69
22	776737777760771	New	Euro-American		T1	0.99	2	3.39
23	777317347760671	New	Euro-American		T1	0.99	1	1.69
24	777777747420771	New	Euro-American		H3	0.77	1	1.69
25	377000377760771	New	Euro-American		T3	0.99	1	1.69
26	777737747760760	New	Euro-American		T1	0.99	1	1.69
27	703777600003171	New	East-African Indian (CAS)		CAS	0.99	1	1.69
28	703701740003171	New	East-African Indian (CAS)		EAI4	0.933	1	1.69
29	006737777760771	New	Euro-American		T1	0.99	1	1.69
30	511000377760771	New	Euro-American		T3	0.99	1	1.69
31	602343761000200	New	*M. bovis*				1	1.69
							59	100 %

CAS (Central Asian Strain), EAI (East African Indian), H (Haralem), LAM (Latin American), T (ill defined)

### Strain analysis

Spoligotyping of isolates from EPTB cases showed that the 59 isolates display 31 different patterns. Of these 35 (59.3 %) strains could be grouped into 7 different clusters (Table 4). The largest cluster (ST53) comprised 12 (20.3 %) isolates, followed by ST149 (10.1 %) and ST25 (8.47 %) with 6 and 5 isolates, respectively. ST910 and ST21 comprised of 3 (5.8 %) isolates for each. The rest were unique (non-clustered) spoligotype patterns in 24 (40.7 %) of the isolates.

**Fig. 3:**
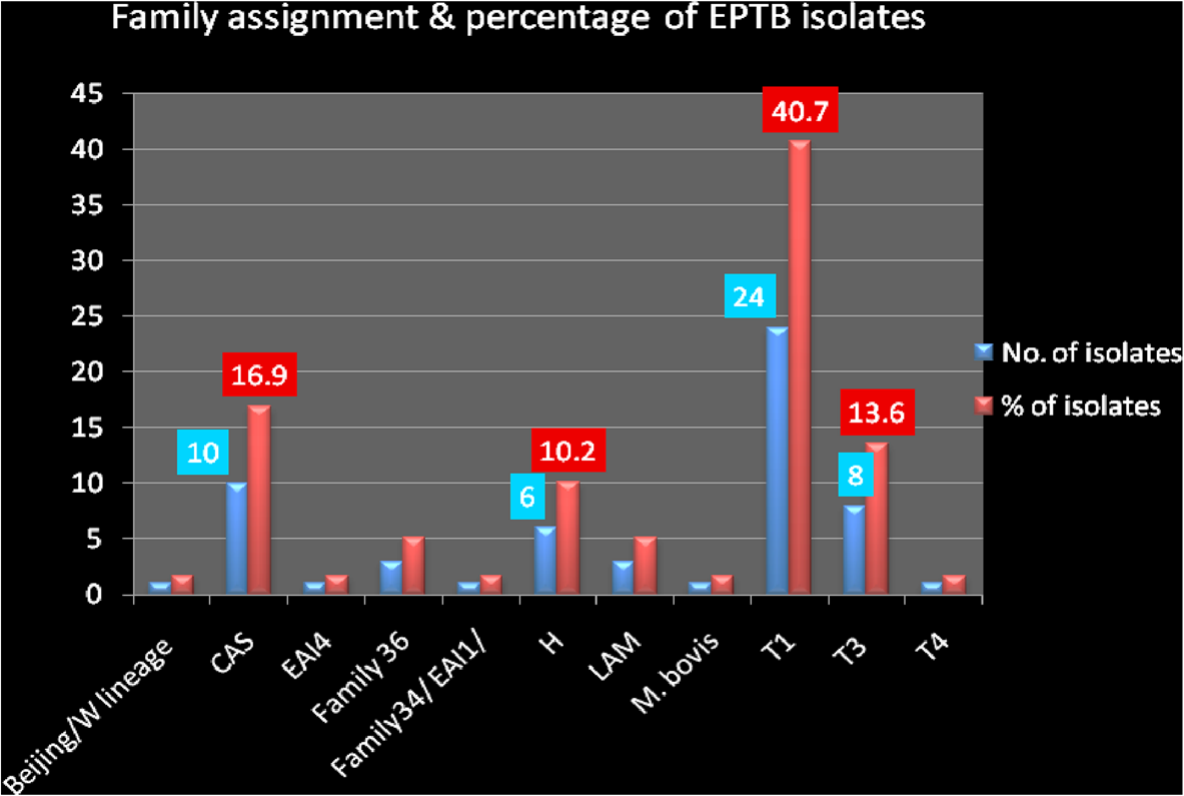
Percentage of Family types of EPTB isolates

All the 59 EPTB isolates (both the new and isolates found in the data base) were assigned in to families and sub lineage using SpolDB4 for the known and SpotClust ID for the new isolates. Hence, the result showed that the T1 lineage is the dominant followed by CAS, T3 and Harlem with a percentage of 40.7, 16.2, 13.6 and 10.2 respectively (Fig. 3).

**Fig. 4:**
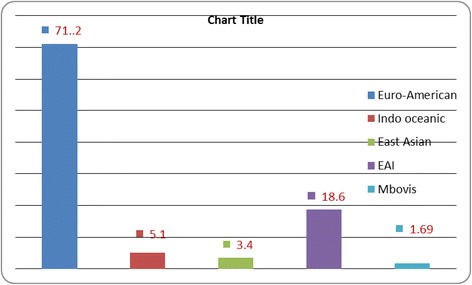
Percentage distribution of Major lineages

In addition, the isolates were also classified into lineage and the result revealed that 69.5 % of the isolates were Euro American lineage followed by East African Indian (CAS) and Indo-Oceanic with 20.3 % and 5.1 % respectively. The rest 5.1 % comprised of Beijing, East Asian and *Mycobacterium bovis* each having one isolate (Fig. 4).

Dendrogram built from 59 *M. tuberculosis* and one *M. bovis* Samples based on spoligotyping results using Unweighted Pair Group Method with Arithmetic mean (UPGMA). Cophenetic correlation coefficient of the dendrogram is 0.827.

**Fig. 5:**
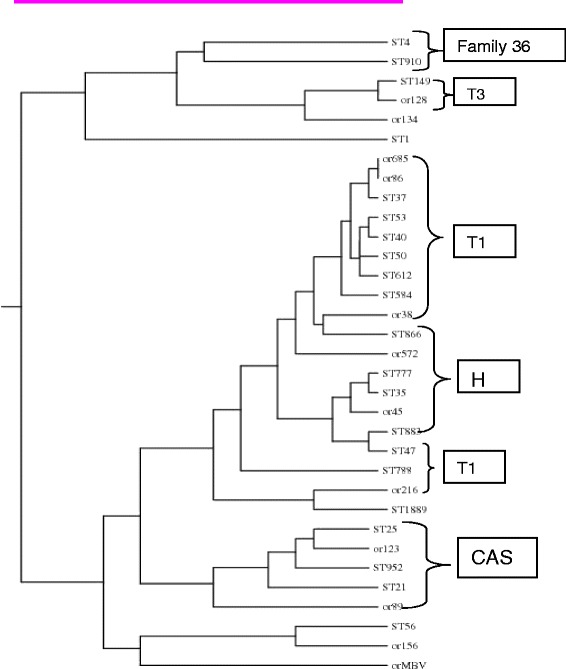
Dendrogram of extrapulmonary tuberculosis isolates based on genetic similarity of DR regions. CAS= Central Asian strain, T= ill-defined lineage, H= Haarlem lineage

The genetic distance was built using the unweighted pair group method with arithmetic mean (UPGMA) algorithm [30] and it showed that the strains clustered in to families: T1, T3, H and CAS. The distance of the measurement is 1 (Fig. 5).

### Relationship between infection sites and isolates family

**Fig. 6:**
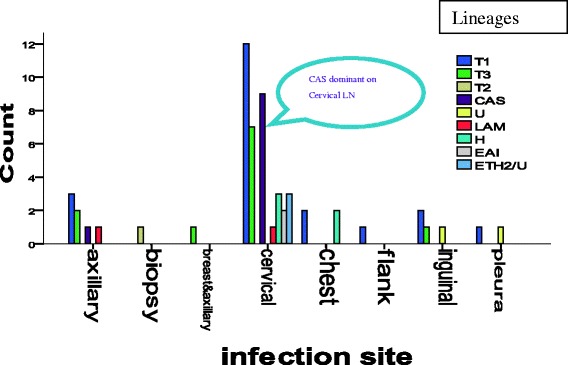
Distribution of the different families at the collection sites, p= 0.889

Since samples were taken from different infection sites, we analysed to see whether there is an association between the sites and the family of the isolates or not. Accordingly, the chi square test showed that there is no relationship between infection site and family/strain types (P = 0.889). However, the CAS strain is found mostly in lymphadenitis: 9 from cervical and 1 axillary (Fig. 6).

### Drug sensitivity

Anti-Mycobacterial Drug sensitivity test for both first and second line drugs: INH, RIF, EMB and STM, revealed that the proportion of resistance to rifampicin was higher (22 %) and that of INH, STM and EMB were 8.1 %, 5 % and 3 % respectively. Out of the 37 isolates tested for resistance, only 2 isolates were resistant for both STM and INH and no MDR was found (Table 5).

**Table 5:** Drug sensitivity of EPTB isolates in percent

	INH	RIF	EMB	STM	INH &STM	INH, EMB & STM
Resistant	3 (8 %)	8 (21.6 %)	1 (2.7 %)	2 (5.4 %)	1 (2.7)	1 (2.7 %)
Susceptible	34 (92 %)	29 (78.4 %)	36 (97.3 %)	35 (94.6 %)	36 (97.3 %)	36 (97.3 %)

## Discussion

Before the beginning of the HIV epidemic, ∼85 % of reported tuberculosis cases were limited to the lungs [31]. Latter, this proportional distribution was considerably different among persons with HIV infection and then EPTB came out of the shadow and becoming a growing problem [29]. At present, Extra-pulmonary tuberculosis is on the increase globally including Ethiopia [32, 33]. The global increase is believed to be increased by HIV related immune incompetence. In addition to this global picture, the national report also shows that it covers almost one third of the total TB case reports [33].

In agreement to these observations, our result showed that among the total suspected cases, 62 (31 %) were HIV positive. We have also found that 21 (35 %) were HIV positive from culture confirmed EPTB cases. However, this figure is higher and accounts for 53 to 62 % in HIV co-infected individuals whereas only 15 to 20 % of all forms of TB in HIV negative patients [34, 35]. Different studies indicated that EPTB increased significantly with the pandemic of HIV and the co-infection rate is higher in developing countries where the prevalence is higher. It has been well established that EPTB is higher among HIV positives. Earlier reports of Abate *et al.* (2003) from Tikur Anbessa hospital in Addis Ababa showed that the proportion of newly diagnosed EPTB patients among HIV patients was more than those of smear positive and smear negative pulmonary TB [36] and other studies also showed that HIV is associated with Extra pulmonary tuberculosis [37, 38]. HIV, poor living condition and inadequate health care contribute to the high level of TB in developing countries (Fanning 1999; Sharma and Mohan, 2004).

The higher proportions of HIV within the age range 25–35 and the higher frequency of HIV and EPTB in female patients was also higher than in males. As suggested in other studies, this might be due to differential exposure of women to infectious TB patients, and medical care compared with men [6, 39].

EPTB has different manifestations based on the organ affected and the distribution varies in different studies. Mohammed *et al*., (2003) reported that lymph node tuberculosis constituted 40 % of all TB cases and 72.8 % of the clinical suspects in Southern Ethiopia [40]. In agreement to the above observations, in our study lymphadenitis, disseminated TB and Pleural TB were the three major types. As shown in Fig. 1, 65 % of the confirmed cases were lymph node tuberculosis including axillary, inguinal and cervical. This is also similar to the study reported from Germany [5]. In contrary, the most common sites involved were the bone/joints and lymph nodes in the United States [4], while the genitourinary system and skin were the common sites in a report from Hong Kong [41]. These differences may be attributable to either host or pathogen related factors as well as access to patient sampling in the clinical settings. Though, strong statistical association is not established, our study showed that there is dominance of certain strains (Fig. 5) and clusters of CAS found only lymphadenitis (Fig. 6).

Analysis of the diversity of Mycobacterial isolates and data base comparison is essential in determining the distribution of *M. tuberculosis* complex in different geographical setting and worldwide. It also gives us an insight to understand the dynamics of current transmission and dominant strains associated with the disease pattern. In line with this, we described the strain diversity of 59 *M. tuberculosis* isolates from extra pulmonary tuberculosis patients in Addis Ababa.

There is geographical sub structuring within the Euro-American lineage and most other areas were also associated with only one or two lineages. However, all six main lineages were represented in Africa [42]. In addition, there is an observation of Indo-Oceanic lineage, the most ancestral of the six lineages, which was associated with East Africa. In agreement to this justification, our isolates from EPTB cases were categorized in to five lineages with the absence of *Mycobacterium africanum* and *M. caprae*. Accordingly Euro American lineage (71.1 %), Indo Oceanic (5.1 %), East Asian (3.4 %) East Africa Indian (18.6 %) and *M. bovis* (1.6 %) (Fig. 4).

In this study, the distribution of strains showed that the T1 family is the dominant followed by CAS, T3 and Harlem with a percentage of 40.7, 16.2, 13.6 and 10.2 respectively. In line with this finding it was reported that the T family and other which were categorized under Euro-American and Indo-Oceanic were predominantly found in extra pulmonary cases [43] and also 42 % of isolates from tuberculous lymphadenitis were belonging to the Harlem genotype [44].

The data base comparison using SpolDB4 to assign into Shared International Number, spoligotyping pattern showed that the largest cluster, ST53 comprised 12 (20.3 %) isolates, followed by ST149 (10.1 %) and ST25 (8.47 %) with 6 and 5 isolates, respectively. ST910 and ST21 comprised of 3 (5.8 %) of isolates for each. The rest were unique (non-clustered) spoligotype patterns in 24 (40.7 %) of the isolates. In addition, all the spoligotypes not found in SpolDB4 data base (orphans), were also assigned to families and sub lineage using SPOTCLUST ID web based algorithm model SpolDB3 (http://tbinsight.cs.rpi.edu/run_spotclust.html). Accordingly most of the new strains obtained from extra-pulmonary cases were assigned to T1 family with a probability of 0.99. Based on these assignments, the T1 lineage was dominant and accounted for 21 (35 %). This might be due to clonal association and an active transmission of this family with in the society as it has been indicated on a number of research it is an indication of active transmission in the community [45–49].

Evidence suggests that there is strain-specific difference in virulence and immunogenicity. Moreover, strain variation might be attributed to the variation in clinical appearances and some strains are more virulent and possess greater ability to disseminate than others [50]. In this study, the isolates were categorized into different sub lineages. Accordingly, the T1, H, CAS, ETH2/U and T3 cover more than 60 % of the EPTB isolates; the T1 is the dominant, widely distributed and isolated from different parts of the body whereas CAS strains are clustered with close similarity and isolated from cervical and axillary samples only. Out of the total 59 isolates 10 were categorized under CAS1 family, of these 9 were found in lymph node tuberculosis. Showing there is dominance of CAS1 high in TBLN. In line with this, studies from Pakistan showed that these strains were also prevalent and accounted for 39 % of isolates form both pulmonary and extra pulmonary cases [51]. Similarly, Lari et al. (2009) reported that there is association between the CAS strains and extra-pulmonary TB [52] which might in part explained by the long established link of extra-pulmonary tuberculosis TB with South Asian of African origin patients that has been also reported earlier. On the other hand, two strain families, LAM3/F11 and W-Beijing, predominated in the South African study [53]. However, in our study we found only a single isolate of Beijing strains which is similar to studies conducted on the same area [54].

Earlier study conducted by Abate etal, (1998) on retreatment cases in Addis Ababa showed that drug resistance of pulmonary tuberculosis to one or more drugs was 50 % and that of MDR TB was 12 % for that period [36] Nevertheless, the overall primary drug resistance to pulmonary tuberculosis was 15.6 % [55] and 21 % [56] in studies conducted in Addis Ababa. According to the national survey (2003–2006), the percentage of MDR TB in Ethiopia is 1.6 and 11.8 of new and retreatment cases respectively. Similarly WHO reported that the % of new and retreatment cases with MDR-TB were 1.6 and 12 respectively [57].

Moreover studies revealed that the frequency of single drug resistance in Extra pulmonary tuberculosis was higher (13.3 %) and that of multiple drug resistance was 15.9 % [58] which is much higher than that reported by the World Health Organization (3.6 %) [59]. Our study showed that the proportion of resistance to rifampicin (21.6 %) was higher and single drug resistance was lower than the above study. However, another study conducted in the eastern part of Ethiopia reported that 9.5 % resistance against isoniazid and the rate of MDRTB was 1.1 % [60]. An additional study also reported that the proportion of resistance to Rifampicin (RMP) and isoniazid were 17 (2.8 %) and 15 (2.5 %) respectively [61]. However, the resistance in our study to other first line drugs were comparatively similar to these studies.

## Conclusion

The study showed that the disseminated and lymphoid tuberculosis is the most frequent forms of extra pulmonary tuberculosis in Addis Ababa. Development of drug resistance in the study isolates was low except for rifampicin and multi drug resistance was not found in the study strain.

The Euro American and East African Indian Lineages are the dominant lineage and SIT 53 strains were also. It has also indicated that CAS appeared to be highly clustered in TBLN than in other forms. A moderate level of cluster formation suggested the presence of recent transmission of TB in the area. The dominance and distribution of the T lineage might be delay in diagnoses of EPTB cases, which will create a condition for dissemination or might suggest higher virulence of these strains as compared to others. Whereas, the localization of CAS strain within cervical areas needs further investigation.
